# Spectral analysis of multiple scattering factors of turbid media for glucose measurement using near-infrared spectroscopy

**DOI:** 10.1117/1.JBO.28.6.065005

**Published:** 2023-06-15

**Authors:** Lu Yue, Han Tongshuai, Liu Wenbo, Ge Qing, Liu Jin

**Affiliations:** Tianjin University, State Key Laboratory of Precision Measuring Technology and Instruments, Tianjin, China

**Keywords:** non-invasive glucose measurement, near-infrared diffuse reflectance spectroscopy, effective attenuation coefficient, Mie scattering theory, differential absorbance, Monte Carlo simulation

## Abstract

**Significance:**

Near-infrared (NIR) diffuse reflectance spectroscopy has been widely used for non-invasive glucose measurement in humans, as glucose can induce a significant and detectable optical signal change in tissue. However, the scattering-dominated glucose spectrum in the range of 1000 to 1700 nm is easily confused with many other scattering factors, such as particle density, particle size, and tissue refractive index.

**Aim:**

Our aim is to identify the subtle distinctions between glucose and these factors through theoretical analysis and experimental verification, in order to employ suitable methods for eliminating these interferences, thus increasing the accuracy of non-invasive glucose measurement.

**Approach:**

We present a theoretical analysis of the spectra of 1000 to 1700 nm for glucose and some scattering factors, which is then verified by an experiment on a 3% Intralipid solution.

**Results:**

We found that both the theoretical and experimental results show that the effective attenuation coefficient of glucose has distinct spectral characteristics, which are distinct from the spectra caused by particle density and refractive index, particularly in the range of 1400 to 1700 nm.

**Conclusions:**

Our findings can offer a theoretical foundation for eliminating these interferences in non-invasive glucose measurement, aiding mathematical methods to model appropriately and enhance the accuracy of glucose prediction.

## 1 Introduction

Diabetes is a metabolic disease characterized by high blood glucose levels, making it one of the most serious chronic diseases in the world.^1^^–^^3^ Among the non-invasive glucose measurement methods, near-infrared (NIR) diffuse reflectance spectroscopy has been well developed with a variety of devices and research results.^4^ The living skin’s blood glucose affects NIR diffuse light intensity by changing the refractive index^5^ and absorption of interstitial fluid, thus the measured diffuse light intensity can monitor the glucose in interstitial fluid, which is highly correlated with the glucose in the blood. In addition, the skin’s scattering also changes when the refractive index of interstitial fluid is altered. Therefore, glucose affects both the absorption and scattering of the skin tissue.^6^^,^^7^

In this paper, we discuss the NIR diffuse light signals of glucose in the 1000 to 1700 nm band. The measured light signal combines the absorption effect and the scattering effect. The scattering can influence the whole waveband of 1000 to 1700 nm, but the absorption mainly affects the signal of 1400 to 1700 nm. In 1400 to 1700 nm, glucose has relatively obvious absorption (mainly caused by O─H bonds and C─H bonds). However, for the 1000 to 1400 nm band, the absorption is very weak and almost none, so the glucose’s response is dominated by scattering. Moreover, even for 1400 to 1700 nm, the glucose-caused absorption coefficient change is much smaller than the change in scattering coefficient, thus the scattering still dominates the glucose signal here.^8^^,^^9^ According to the study of Amerov et al., the absorption coefficient in aqueous solution can change by $∼0.00065\;{\mathrm{cm}}^{-1}$ at the wavelength range of 1500 to 1600 nm for every 1 mM increase in glucose, whereas it is almost zero for 1000 to 1300 nm.^10^ According to Liu et al., the reduced scattering coefficient at 1000 nm will be approximately $-0.008\;{\mathrm{cm}}^{-1}$ for 1 mM glucose, which is much greater than the change in absorption coefficient.^11^ According to optical coherence tomography (OCT) results, the scattering coefficient change caused by glucose in living tissue is significantly greater than that in phantom solutions, and the coherent signal response caused by glucose in living tissue can reach tens or hundreds of times that of phantom solution.^12^ In this paper, we discuss the contributions of absorption and scattering of glucose, respectively, to the glucose signal in Intralipid solution.

Except for glucose, many other factors, such as the ions (${\mathrm{Na}}^{+}$, $K^{+}$, etc.) in the interstitial fluid, the density of tissue particles, and the size of tissue particles, can affect tissue scattering and lead to similar spectra to the glucose’s scattering spectrum, thus reducing the accuracy of glucose prediction.^13^ Unfortunately, chemometrics methods, such as partial least squares regression,^14^ still have no good solution for this problem.

In fact, there are slight differences between the spectra of the above scattering variables. The concentrations of ions (${\mathrm{Na}}^{+}$, $K^{+}$, etc.) and glucose in the interstitial fluid affect the fluid’s refractive index; the density and size of particles in tissue can change the particles’ distribution. Both the fluid’s refractive index and the particles’ distribution can change the scattering coefficient of tissue.^15^ In addition, glucose has additional absorption in the NIR band, particularly in the range of 1400 to 1700 nm. This paper aims to analyze the spectral differences between the above factors, in order to provide theoretical support to distinguish glucose from other factors, especially in terms of finding reasonable wavelengths.

We use the theory of Mie scattering to discuss the scattering difference between the factors.^16^^,^^17^ Mie scattering theory is often based on the equations proposed by Van.^18^ According to Van’s theory, Graaff et al. obtained the approximate calculation formula of the reduced scattering cross section using the relative refractive index, particle size, and particle density, thus promoting the application of Mie scattering theory in biological tissues.^19^ Kohl et al. provided a simplified equation of the reduced scattering coefficient for tissues to estimate the effect of glucose on tissue scattering.^20^ Liu et al. investigated the scattering alteration of Intralipid solution and *ex vivo* tissue when carbohydrate was added to the media.^21^

In the 1000 to 1700 nm range, we have selected six significant wavelengths for glucose measurement, namely 1050, 1219, 1314, 1409, 1550, and 1609 nm. Glucose absorption is minimal at 1050, 1219, and 1314 nm, mainly used to reflect glucose scattering, whereas 1409, 1550, and 1609 nm provide more apparent absorption information for glucose and produce comprehensive results of glucose absorption and scattering.^4^ The 1050 nm wavelength can exhibit hemoglobin’s absorption^22^ and can be used to monitor blood flow. Fat has strong absorption at 1219 nm,^23^ which can reflect changes in subcutaneous fat. Water has higher absorption at 1409 nm, so it is greatly influenced by skin water content and temperature.^24^ In addition, 1314 nm is a compromise wavelength that is relatively insensitive to hemoglobin, fat, water, and even temperature changes.^25^^,^^26^ Therefore, multiple wavelengths are necessary to acquire enough information. Han et al. reported the glucose measurement results using the six wavelengths on human subjects,^27^ which showed that the 1409, 1550, and 1609 nm wavelengths exhibit excellent glucose measurement sensitivity.

The source–detector separations (SDSs) selected in this paper are also reasonable. Liu et al. discovered that there are corresponding suitable SDS ranges for different wavelengths, where ΔA shows an approximately linear change with SDS.^11^ For shorter wavelengths, SDS can be 0.8 to 5 mm.^28^^,^^29^ The SDSs used in this paper, 1.25 to 2.3 mm, can meet the measurement requirements of the six wavelengths. The small SDSs of 1.25 to 1.7 mm could be better than other SDSs for 1409, 1550, and 1609 nm.

## 2 Theoretical Analysis of Scattering Factors

### 2.1 Reduced Scattering Coefficient in Mie Scattering Theory

According to Graaff’s study,^19^ when the wavelength λ and particle’s radius a meet the case of 5<2πa/λ<50, the reduced scattering cross-section $\sigma_{s}^{\prime }$ can be approximated as

$$\sigma_{s}^{\prime }=\sigma_{s}(1-g)=3.28\pi a^{2}{\left(\frac{2\pi a}{\lambda }\right)}^{0.37}{\left(m-1\right)}^{2.09},$$ (1)

where $\sigma_{s}$ is the scattering cross-section, g is the anisotropy factor, $n_{\mathrm{in}}$ is the refractive indices inside the particles, $n_{\mathrm{ex}}$ is for that outside the particles, i.e., the refractive indices of the solution in the tissue, and $m=n_{\mathrm{in}}/n_{\mathrm{ex}}$ is the relative refractive index.

When the scatterer is spherical, the reduced scattering coefficient of tissues can be expressed as^30^

$$\mu_{s}^{\prime }=\frac{3\phi }{4\pi a^{3}}\sigma_{s}^{\prime }=\frac{2.46\phi }{a}{\left(\frac{2\pi a}{\lambda }\right)}^{0.37}{\left(m-1\right)}^{2.09},$$ (2)

where ϕ is the volume fraction of the scattered particles.

**Fig. 1:**
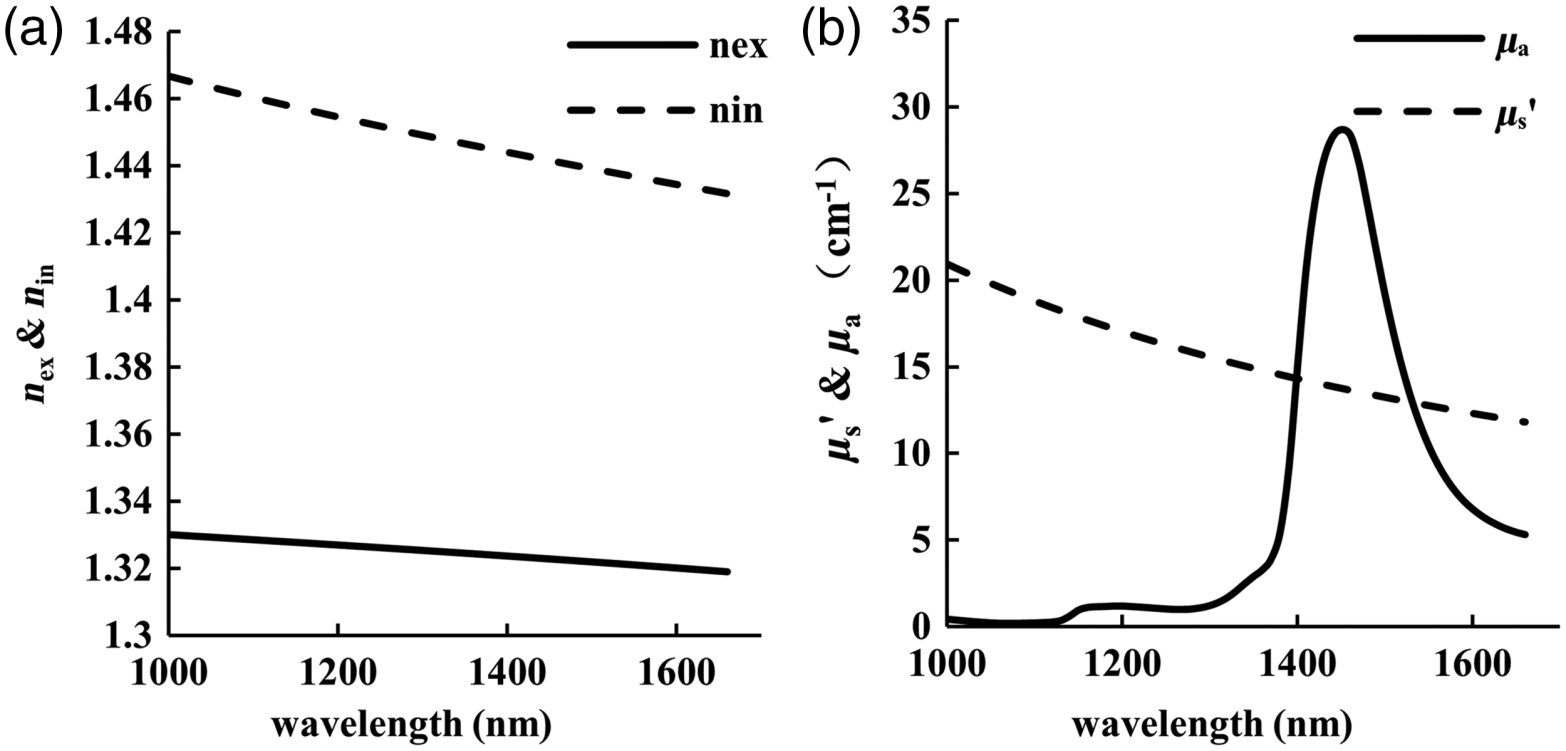
Optical properties of 3% Intralipid solution: (a) The refractive index and (b) absorption coefficient and reduced scattering coefficient.

In a 3% Intralipid solution, soybean oil particles (oil) are the main scattering particles, with the background solution being predominantly aqueous.^31^^–^^33^ The volume fraction of scattered particles (soybean oil) was about 0.03237. Christian et al. and Xu et al. determined the average particle size of the Intralipid solution is ∼1.00±0.14  μm.^34^^,^^35^ In the 1000 to 1660 nm band, the $n_{\mathrm{in}}$ and $n_{\mathrm{ex}}$ of Intralipid solution are shown in Fig. 1(a).^36^

Using the typical values of a (1.00  μm), ϕ (0.03237), $n_{\mathrm{in}}$, and $n_{\mathrm{ex}}$, the reduced scattering coefficient can be calculated by using Eq. (2), as shown in Fig. 1(b). The absorption coefficient of the solution also is shown in Fig. 1(b).

### 2.2 Scattering Change Caused by Solution’s Refractive Index $n_{\mathrm{ex}}$

According to Eq. (2), the derivative of the scattering coefficient with respect to the background refractive index $n_{\mathrm{ex}}$ can be as

$$\frac{d\mu_{s}^{\prime }}{dn_{\mathrm{ex}}}=-\frac{5.1414\phi }{an_{\mathrm{ex}}^{3.09}}{\left(\frac{2\pi a}{\lambda }\right)}^{0.37}\cdot n_{\mathrm{in}}\cdot {\left(n_{\mathrm{in}}-n_{\mathrm{ex}}\right)}^{1.09}=-2.09\mu_{s}^{\prime }\frac{n_{\mathrm{in}}}{n_{\mathrm{ex}}^{2}}{\left(m-1\right)}^{-1}.$$ (3)

**Fig. 2:**
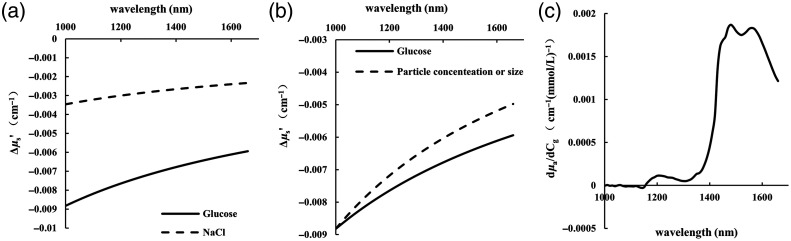
The changes of absorption coefficient and scattering coefficient caused by four variables in 3% Intralipid solution. (a) $d\mu_{s}^{\prime }/dC_{g}$ and $d\mu_{s}^{\prime }/dC_{\mathrm{NaCl}}$; (b) $\Delta \mu_{s}^{\prime }$ caused by 0.00137% volume fraction change (or particle size change 0.336 nm) and 1 mM glucose; (c) $d\mu_{a}/dC_{g}$.

Using Eq. (3), we can estimate the $d\mu_{s}^{\prime }/dC_{g}$ and $d\mu_{s}^{\prime }/dC_{\mathrm{NaCl}}$, i.e., $\Delta \mu_{s}^{\prime }$ caused by 1 mM glucose concentration ($C_{g}$) and 1 mM ion concentration ($C_{\mathrm{NaCl}}$, as we use NaCl), where $dn_{\mathrm{ex}}/dC_{g}$ for aqueous solution is set to about $2.5\times 10^{-5}\;{\mathrm{mM}}^{-1}$, and $dn_{\mathrm{ex}}/dC_{\mathrm{NaCl}}$ for aqueous solution is set to about $0.98\times 10^{-5}\;{\mathrm{mM}}^{-1}$.^37^^,^^38^ Figure 2(a) shows the results.

### 2.3 Scattering Change Caused by Particle Density

According to Eq. (2), the derivative of the scattering coefficient with respect to the particle density (i.e., volume fraction ϕ) can be as

$$\frac{d\mu_{s}^{\prime }}{d\phi }=\frac{2.46}{a^{0.63}}{\left(\frac{2\pi }{\lambda }\right)}^{0.37}{\left(m-1\right)}^{2.09}=\mu_{s}^{\prime }\frac{1}{\phi },$$ (4)

where the volume fraction of scattered particles ϕ in 3% Intralipid solution is about 0.03237.

### 2.4 Scattering Coefficient Change Caused by Particle Size

According to Eq. (2), the derivative of the scattering coefficient with respect to the particle size (i.e., particle radius a) can be as

$$\frac{d\mu_{s}^{\prime }}{da}=-\frac{1.5498\phi }{a^{1.63}}{\left(\frac{2\pi }{\lambda }\right)}^{0.37}{\left(m-1\right)}^{2.09}=-0.63\mu_{s}^{\prime }\frac{1}{a}.$$ (5)

The radius of the particles in Intralipid solution is ∼0.50±0.07  μm. The average particle radius of 0.50  μm is used in this paper.

Our analysis shows that the $\Delta {\mu_{s}}^{\prime }$ caused by particle concentration and particle size are contained within the previous coefficients, which remain identical when ΔΦ is 0.00137% and Δa is 0.336 nm. At 1000 nm, their values are the same as those caused by 1 mM glucose, but they differ at other wavelengths: the absolute value of $\Delta {\mu_{s}}^{\prime }$ caused by glucose is slightly larger than that caused by particle density or size. These findings are presented in Fig. 2(b).

## 3 Differential Absorbance

According to the first-order approximation of the radiative transport equation, for a semi-infinite medium, at a SDS of r, the diffuse reflection light intensity can be expressed as

$$I(r)=I_{0}\frac{1}{4\pi }[z_{0}(\mu_{\mathrm{eff}}+\frac{1}{r_{1}})\frac{e^{-\mu_{\mathrm{eff}}r_{1}}}{r_{1}^{2}}+(z_{0}+2z_{b})(\mu_{\mathrm{eff}}+\frac{1}{r_{2}})\frac{e^{-\mu_{\mathrm{eff}}r_{2}}}{r_{2}^{2}}],$$ (6)

where $I_{0}$ is the incident light intensity, $\mu_{\mathrm{eff}}$ is the effective attenuation coefficient (EAC), $\mu_{\mathrm{eff}}=[3\mu_{a}(\mu_{a}+\mu_{s}^{\prime }){]}^{1/2}$, D is the diffusion coefficient, $D=1/3(\mu_{a}+\mu_{s}^{\prime })$, $r_{1}=\sqrt{r^{2}+z_{0}^{2}}$, $r_{2}=\sqrt{r^{2}+(z_{0}+2z_{b}{)}^{2}}$, $z_{0}=1/\mu_{s}^{\prime }$, and $z_{b}=2D$.

The diffuse light absorbance A at the SDS of r is defined by the natural logarithm as

$$A(r)=-\ln\;\frac{I(r)}{I_{0}}.$$ (7)

Differential absorbance $A_{D}$ between two SDSs ($r_{A}$ and $r_{B}$) can be

$$A_{D}=A(r_{B})-A(r_{A})=\ln\frac{I(r_{A})}{I(r_{B})},$$ (8)

$$A_{D}\approx \mu_{\mathrm{eff}}(r_{A}-r_{B}).$$ (9)

For the given SDSs $r_{A}$ and $r_{B}$, differential absorbance is approximated as the variable related to EAC,^39^ which can reduce the light source drift, instrument drift, etc. Thus, the change in EAC can be approximated as

$$\Delta \mu_{\mathrm{eff}}=\Delta A_{D}/(r_{A}-r_{B}).$$ (10)

The above derivation roughly explains the characteristic that absorbance change ΔA shows an approximately linear change with SDS when the component changes. The above derivation may not be suitable for wavelengths near 1450 nm with too strong water absorption and SDSs too close or too far away. Nevertheless, the characteristic that absorbance ΔA shows a monotonic change with SDS will remain unaffected, and its non-linearity can be corrected as a systematic error in measurement.

## 4 Monte Carlo Simulation

The MCMLGO program written by Lihong V. Wang was used to simulate the diffuse reflection intensity of the scattering media (3% Intralipid solution). The number of photons was set to $10^{14}$, and the wavelengths are 1000 to 1660 nm with an interval of 10 nm. The media’s optical parameters ($\mu_{a}$, $\mu_{s}$, n, g) were set according to Fig. 1(b), and their changes $\Delta \mu_{a}$, $\Delta \mu_{s}^{\prime }$ and Δn caused by glucose, NaCl, and Intralipid solution concentration were set according to Fig. 2. (1) The first group includes the solutions with 0 and 100 mM glucose. $dn/dC_{g}$ was set to $2.5\times 10^{-5}\;{\mathrm{mM}}^{-1}$; Furthermore, glucose-induced $\Delta \mu_{a}$ and $\Delta \mu_{s}^{\prime }$ were independently set in two simulations in order to illustrate their separate effects. (2) The second group simulates the diffuse reflection light intensity of the solution with 100 mM NaCl, $dn/dC_{\mathrm{NaCl}}$ is $0.98\times 10^{-5}\;{\mathrm{mM}}^{-1}$. (3) The third group simulated the diffuse reflection intensity of the two Intralipid solutions (3%, 3.5%).

**Fig. 3:**
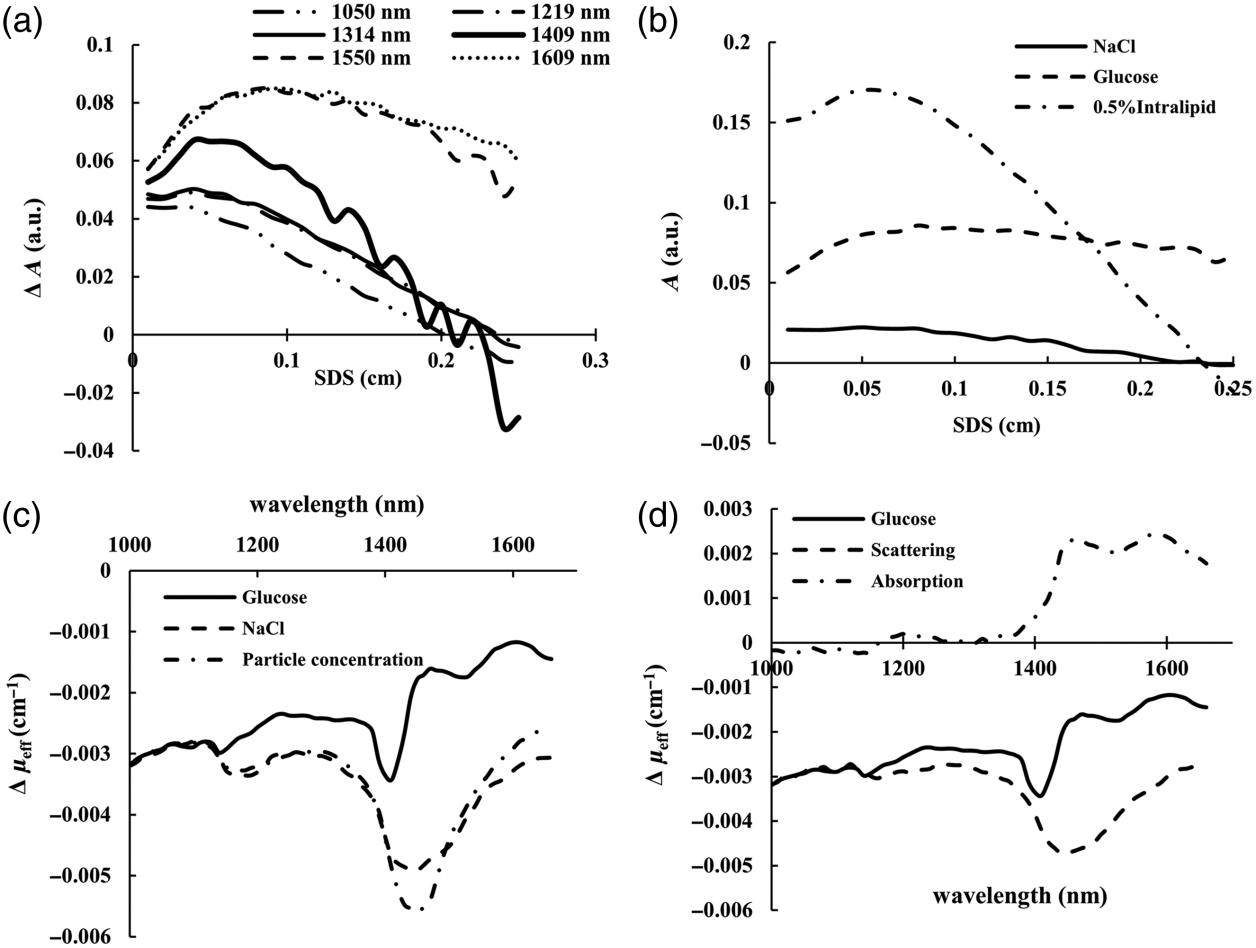
MC simulation results. (a) ΔA of SDSs of 0.01 to 0.25 cm caused by 100 mM glucose at six wavelengths; (b) ΔA of SDSs of 0.01 to 0.25 cm caused by 100 mM glucose, 100 mM NaCl, and 0.5% Intralipid solution concentration (1600 nm); (c) $\Delta \mu_{\mathrm{eff}}$ caused by 1 mM glucose, 2.68 mM NaCl, and 0.001525% Intralipid solution; (d) $\Delta \mu_{\mathrm{eff}}$ caused by 1 mM glucose, glucose’s scattering, and glucose’s absorption.

According to Eqs. (7) and (8), the absorbance of the SDSs of 0.01 to 0.25 cm for the three groups of simulation can be obtained [Fig. 3(b)], and then the differential absorbance and EAC can be calculated using Eqs. (8) and (9). Two SDSs (0.08 and 0.2 cm) were selected for differential processing. In the first group, the spectrum of 1 mM glucose was estimated from the spectrum of 100 mM glucose, the spectrum of NaCl of 2.68 mM was estimated from the spectrum of 100 mM NaCl, and the spectrum of 0.001525% Intralipid solution concentration was estimated from the data of 0.5% Intralipid solution concentration change (3% and 3.5%). After the data processing, the $\Delta \mu_{\mathrm{eff}}$ caused by 1 mM glucose, 2.68 mM NaCl, and 0.001525% Intralipid solution concentration can be the same at 1000 nm, and the three spectra were compared in Fig. 3(c).

Figure 3(d) shows the $\Delta \mu_{\mathrm{eff}}$ results caused by the absorption change of glucose, the scattering change of glucose, and the two effects together. It is evident that scattering change is the predominant factor in the glucose signal, and glucose absorption can only be observed between 1400 and 1700 nm. Further, $\Delta \mu_{\mathrm{eff}}$ caused by glucose’s scattering and absorption at 1400 to 1700 nm shows the opposite results, then the absolute value of $\Delta \mu_{\mathrm{eff}}$ is reduced here.

## 5 Experiment

### 5.1 Measurement System

**Fig. 4:**
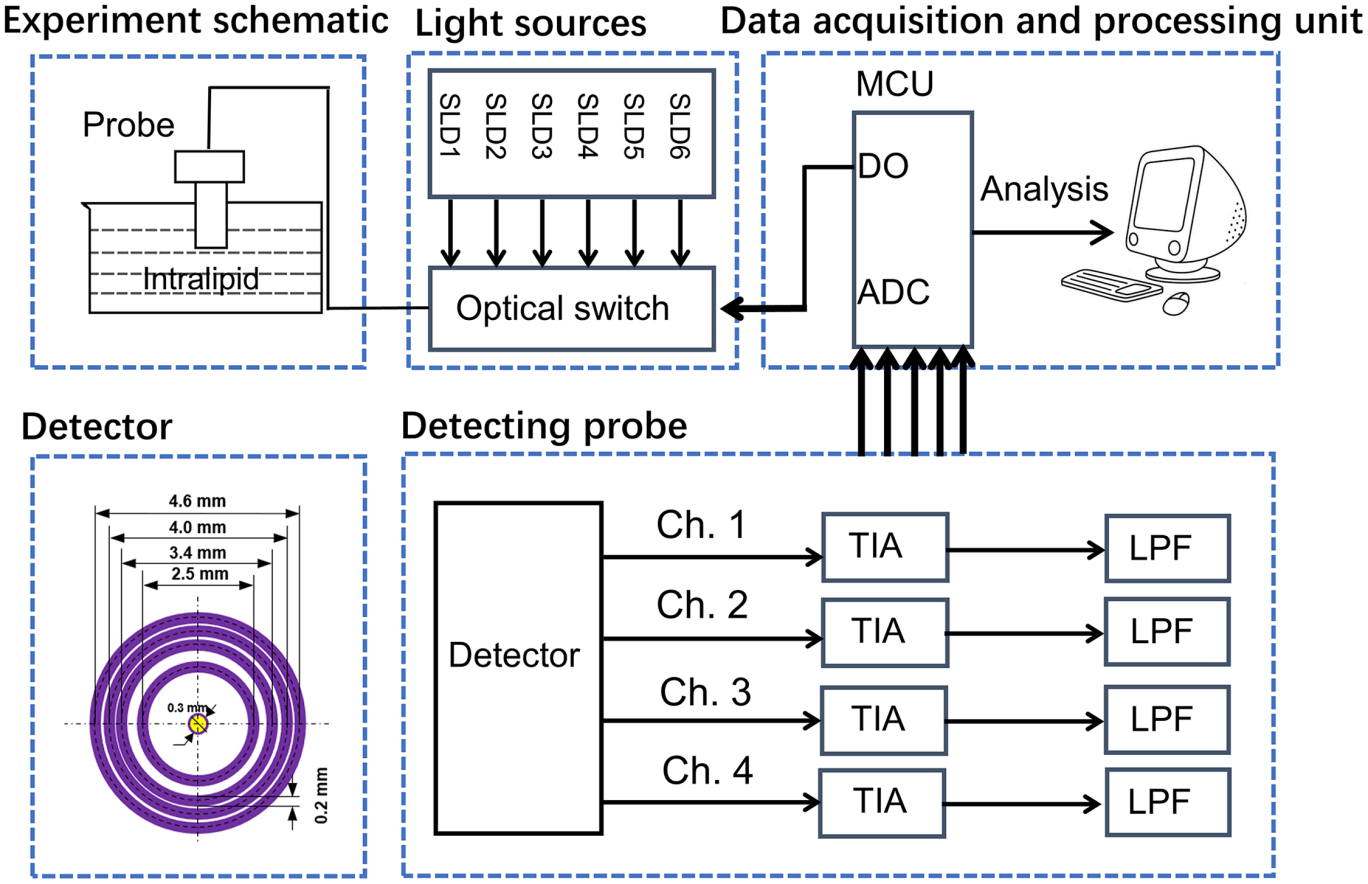
Measurement system diagram.

The measurement system shown in Fig. 4 is composed of a light source, an optical switch, a sensing probe, and a data processing unit. The light source consists of six superluminescent diodes (Inphenix) with center wavelengths of 1050, 1219, 1314, 1409, 1550, and 1609 nm (bandwidth 60 to 130 nm). Data sampling at each wavelength takes ∼3  s. The incident light is guided by a silicon fiber with a core diameter of 105  μm. The photosensitive area of the detector (U-Science Co., Ltd.) is made of a ring-shaped InGaAs material with a width of 0.2 mm. The detector includes four separate photosensitive areas, in the middle of which the SDSs are 1.25, 1.7, 2.0, and 2.3 mm, respectively. Each pair of two adjacent sensing areas is used for differential processing. At the farthest SDS of 2.3 mm, the absorbance fluctuations of six wavelengths within 1 min are respectively ∼0.001, ∼0.002, ∼0.002, ∼0.004, ∼0.002, and ∼0.003 a.u., whereas the fluctuation amplitudes under the other closer SDSs are smaller.

### 5.2 Materials

30% Intralipid solution (Fresenius Kabihuarui Pharmaceutical Co., Ltd.) was used in the experiment. First, the 30% Intralipid solution was diluted to 3% with pure water. Then, the 3% Intralipid solution with glucose and NaCl were prepared, respectively, by adding the anhydrous glucose powder or NaCl powder. The 3.5% Intralipid solution was made by mixing the 3% and 30% Intralipid solution in the proper ratio. For each test, the 3% Intralipid solution was measured first, as the baseline of the other solutions.

### 5.3 Experimental Results

**Fig. 5:**
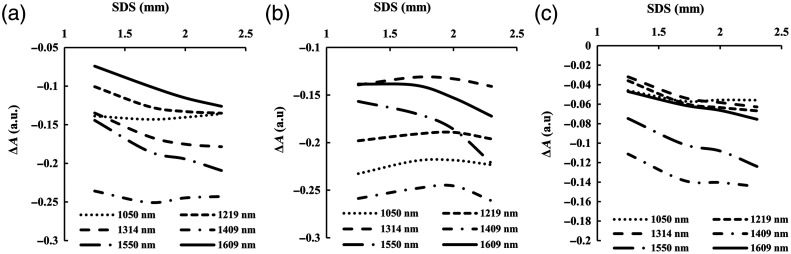
The experimentally measured ΔA changes at the SDSs of 1.25, 1.7, 2.0, and 2.3 mm, respectively. (a) The absorbance caused by 100 mM glucose; (b) the absorbance caused by 200 mM NaCl; and (c) the absorbance caused by 0.5% Intralipid solution concentration change.

**Fig. 6:**
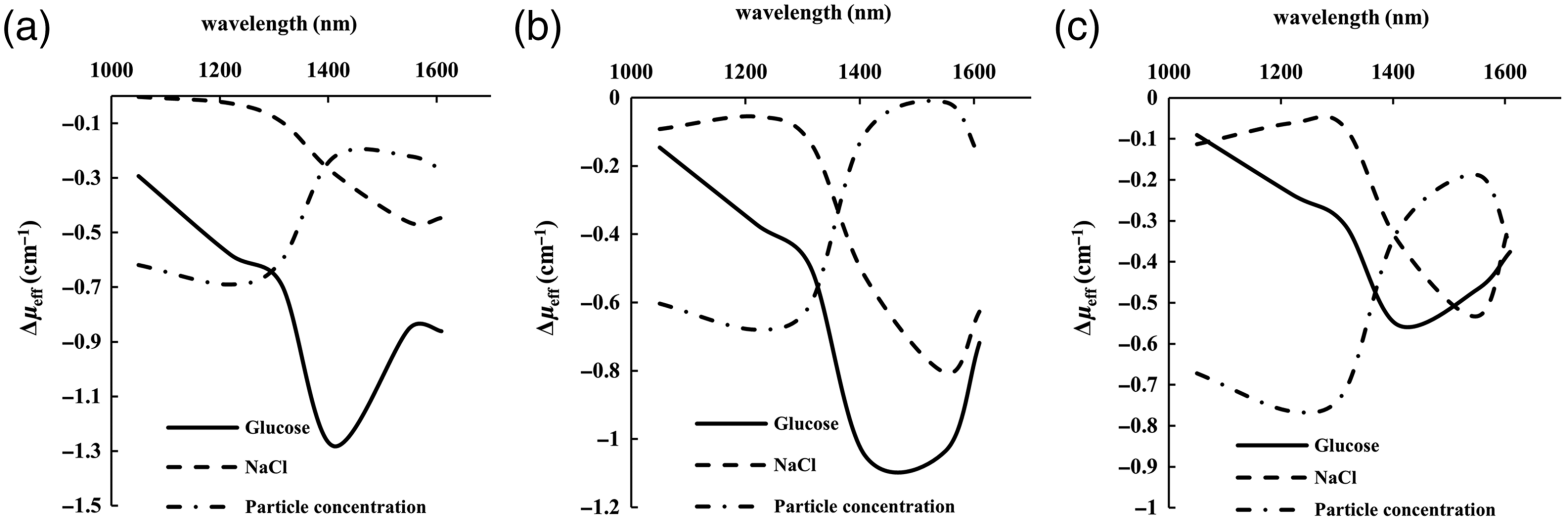
The experimentally measured $\Delta \mu_{\mathrm{eff}}$ changes in the concentration of 100 mM glucose, 200 mM NaCl, and Intralipid solution by 0.1%, respectively. (a) 1.25 and 1.7 mm differential; (b) 1.7 and 2.0 mm differential; (c) 2.0 and 2.3 mm differential.

The diffuse light intensity was recorded at the four SDSs, and the differential absorbance of two adjacent SDSs was obtained using Eqs. (8) and (9). The experimental results are presented in Figs. 5 and 6. The absorbance caused by a 100 mM glucose, 200 mM NaCl, and 0.5% Intralipid solution concentration change at SDSs of 1.25, 1.7, 2.0, and 2.3 mm are shown in Fig. 5. The EAC spectra were calculated using Eq. (10), as shown in Fig. 6. Due to the differences in the coverage and effectiveness of the four photosensitive regions, there are systematic biases in the EAC values.

From Fig. 5, it can be observed that ΔA varies approximately linearly with SDS, but this linearity is not consistent for every wavelength and SDS. The theoretical results of ΔA versus SDS from MC simulation are shown in Fig. 3(a). Overall, the linearity of the three shorter wavelengths (1050, 1219, and 1314 nm) is better than that of the three longer wavelengths (1409, 1550, and 1609 nm), possibly due to the shorter wavelengths being less absorbed by the skin, having longer optical paths, deeper penetration depths, and better adhering to diffusion equation. In a more detailed analysis, the linearity of the three shorter wavelengths is worse for smaller SDSs compared to larger SDSs due to scattering effects, whereas the linearity of the three longer wavelengths deteriorates as SDS becomes too large, and light attenuates sharply due to the stronger absorption of water. Therefore, each wavelength has a suitable linear range of SDS for measurement, neither too close nor too far. Due to limitations in the measurement system and the maximum tolerable light intensity by the skin, it is not possible to infinitely increase the energy of the light sources, resulting in systemic differences between the differential results. The use of different SDSs for differential processing will also produce varying EACs, as demonstrated in Fig. 6. In addition to the aforementioned reasons, other system errors may also exist in the measurement process, such as the angle of incidence of the light source not being completely vertical, leading to eccentricity, and resulting in sensing deviation for each SDS.

As shown in Fig. 6, for a given pair of SDSs, the spectra of the three variables appear different. First, the glucose’s spectra show great differences to the spectra of NaCl and Intralipid solution concentration change since it shows the maximum of $\Delta \mu_{\mathrm{eff}}$ is around 1409 nm, which are more likely around 1550 nm for the spectra of NaCl and Intralipid solution concentration change. Second, the glucose’s $\Delta \mu_{\mathrm{eff}}$ spectra at 1050 to 1314 nm is different from the others, as they always appear a great change with wavelength. However, for the $\Delta \mu_{\mathrm{eff}}$ at 1050 to 1314 nm of NaCl and Intralipid solution concentration change, they are similar or just have little difference between the wavelengths.

In the MC simulation results of Fig. 3, we can also see the differences in glucose and the others. The maximum of glucose’s $\Delta \mu_{\mathrm{eff}}$ also appear around 1400 nm, but the maximums of $\Delta \mu_{\mathrm{eff}}$ caused by NaCl or Intralipid solution concentration are more likely around 1450 nm. Meanwhile, there are differences for the spectra of 1000 to 1300 nm, the glucose spectrum is slightly upward due to the glucose absorption’s effect. It is different from the experimental results, in which the glucose’s spectrum at 1000 to 1300 nm is slightly downward. In our theoretical analysis, the water concentration change was ignored for the particle concentration change and particle size change, thus the experiment may show the true results.

## 6 Conclusion

In this paper, the spectral analysis based on the diffuse equation, Monte Carlo simulation, and measurement experiment was used to investigate the differences between the spectra caused by glucose and other scattering factors in the 1000 to 1700 nm band. The investigated scattering-induced factors include the medium’s refractive index, particle concentration, and particle size. The theoretical analysis using the diffuse equation and Monte Carlo simulation showed that the particle concentration and particle size have similar spectral characteristics. In the experiment, the spectra of glucose concentration, NaCl concentration, and particle concentration in solutions were tested. The NaCl concentration and particle concentration are related to the refractive index change and particle density change, respectively. The two scattering factors showed similar spectrum characteristics in the experiment.

Moreover, we have found that the glucose has definite differences from the other scattering factors both in the theoretical analysis and the experimental results: in the 1000 to 1300 nm band, the spectrum of glucose changes more with wavelength while the spectra of the scattering factors (such as NaCl concentration and particle concentration) are gently here; in the 1400 to 1700 nm band, due to the glucose’s absorption, the glucose’s spectrum increases significantly and the maximum of the spectral curve of the $\Delta \mu_{\mathrm{eff}}$ move to around 1400 nm as it is around the longer wavelength (1450 nm for MC results and 1550 nm for experiment results) for the spectra of NaCl and particle concentration. The above spectral characteristics can be well applied to distinguish the glucose’s spectrum and the spectra caused by the other scattering-induced factors.

In this paper, we explore the combined effects of glucose absorption and scattering in intralipid solution. Our findings could be useful in identifying glucose signals *in vivo*, despite the fact that scattering signals from glucose are much stronger in the human body than *in vitro* or in the Intralipid solution.^15^ To monitor glucose scattering in the human body, wavelengths in the range of 1000 to 1300 nm can be employed, whereas the combined effect of scattering and absorption in the human body can be obtained using wavelengths of 1400 to 1700 nm. Because multiple wavelengths may be necessary for accurate glucose signal identification and multivariate calibration, our results highlight the importance of considering these combined effects when developing new methods for glucose monitoring.
